# CNOT11 depletion is associated with autophagy-related responses and IL-6–JAK–STAT signaling in cancer cells

**DOI:** 10.3389/fcell.2026.1854333

**Published:** 2026-07-17

**Authors:** Saori Nishijima, Toru Suzuki, Tadashi Yamamoto

**Affiliations:** 1 Cell Signal Unit, Okinawa Institute of Science and Technology Graduate University, Okinawa, Japan; 2 Laboratory for Immunogenetics, Center for Integrative Medical Science, RIKEN, Yokohama, Japan

**Keywords:** autophagy-related responses, CCR4-NOT complex (CNOT), CNOT11, IL-6, JAK-STAT signaling, lysosome

## Abstract

**Introduction:**

The CCR4‐NOT complex is a central regulator of deadenylation-mediated mRNA decay, yet the role of its vertebrate-specific subunit CNOT11 remains unclear.

**Methods:**

We investigated the role of CNOT11 in cellular stress responses using siRNA-mediated knockdown, immunoblotting, immunoprecipitation, transcriptomic analysis, quantitative RT-PCR, ELISA, cycloheximide chase assays, actinomycin D treatment, and poly(A) tail analysis.

**Results:**

CNOT11 depletion did not markedly alter the expression of other CCR4‐NOT subunits but reduced the association of CNOT10 with the complex. CNOT11 knockdown was associated with LC3-II accumulation, transcriptional upregulation of autophagy-related genes, and changes in AMPK/ULK1 signaling. Increased IL-6 expression and secretion and enhanced STAT1 and STAT3 phosphorylation were also observed. IL-6 knockdown or STAT3 inhibition partially attenuated LC3-II accumulation. Increased IL-6 expression was associated with elevated transcription, without detectable changes in mRNA stability or poly(A) tail length.

**Discussion:**

These findings suggest that CNOT11 depletion is associated with LC3-II accumulation and other autophagy-related responses, with IL-6‐JAK‐STAT signaling contributing partially to this phenotype. Definitive assessment of autophagic flux and the causal positioning of IL-6 signaling will require further studies using gold-standard flux assays and IL-6 rescue or neutralization approaches.

## Highlights

- CNOT11 depletion is linked to autophagy-related responses in cancer cells
- IL-6/JAK–STAT signaling partially contributes to LC3-II accumulation
- Mitophagy-related transcripts are suppressed after CNOT11 depletion
- IL-6 upregulation is associated with increased transcription

## Introduction

Regulation of gene expression is essential for cellular function, maintenance of homeostasis, and adaptation to environmental changes. mRNA abundance is controlled not only at the transcriptional level by transcription factors but also at the post-transcriptional level through RNA-binding proteins and non-coding RNAs. Increasing evidence highlights the importance of mRNA stability and translational efficiency as key determinants of gene expression, with mRNA degradation pathways playing a central regulatory role. A typical mRNA decay pathway begins with shortening of the poly(A) tail termed deadenylation, which critically influences mRNA abundance and turnover (Mugridge et al., 2018).

The carbon catabolite repressor 4–negative on TATA-less (CCR4–NOT) complex is a conserved multi-subunit protein complex that functions as the principal deadenylase in eukaryotic cells (Collart, 2003). Disruption of CCR4–NOT function leads to widespread alterations in gene expression and has been associated with defects in cellular homeostasis and stress responses. In mammalian cells, the complex comprises multiple subunits organized around the scaffold protein CNOT1 (Ito et al., 2011a; Shirai et al., 2014). CNOT6/6L and CNOT7/8 possess deadenylase activity, whereas CNOT2 and CNOT3 contribute to complex stability (Ito et al., 2011b; Morita et al., 2011; Boland et al., 2013). Additional subunits extend CCR4–NOT function beyond mRNA decay; for example, CNOT9 participates in microRNA-mediated silencing and transcriptional regulation, whereas CNOT10 contributes to the structural organization and functional regulation of the CCR4–NOT complex (Chen et al., 2014; Collart, 2016). *CNOT11* (also known as C2ORF29) is a vertebrate-specific component of the CCR4–NOT complex identified through its association with CNOT10 (Mauxion et al., 2013). Depletion of CNOT11 does not markedly affect global deadenylation kinetics in reporter assays, suggesting that CNOT11 may have functions beyond direct regulation of bulk mRNA deadenylation (Mauxion et al., 2013). Structural analyses have revealed that the CNOT1–CNOT10–CNOT11 submodule provides a structural platform for protein–protein interactions (Mauxion et al., 2023). Furthermore, CNOT11 has been implicated in the regulation of mRNA metabolism, although its precise molecular functions remain unclear.

In this study, we investigated the potential roles of CNOT11 in human cancer cells and found that CNOT11 depletion reduced the association of CNOT10 with the CCR4–NOT complex and was accompanied by autophagy-related responses and cytokine signaling.

## Results

### CNOT11 knockdown selectively affects CCR4–NOT complex association

**FIGURE 1:**
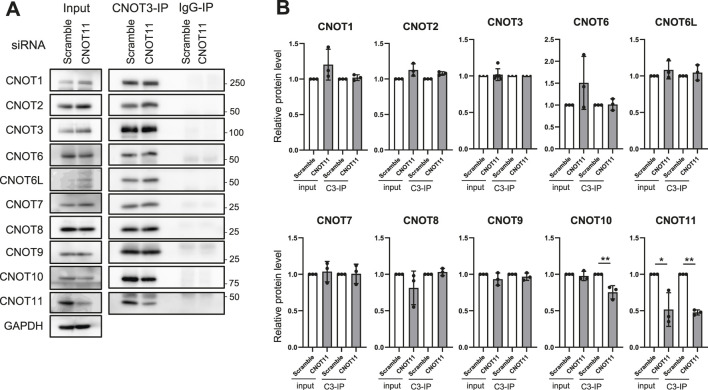
Effects of CNOT11 knockdown on CCR4–NOT subunit association **(A)** HeLa cells transfected with scramble or CNOT11 siRNA were subjected to anti-CNOT3 immunoprecipitation followed by immunoblot analysis with the indicated antibodies. Normal mouse IgG was used as a negative control. **(B)** Quantification of immunoblot data shown in **(A)**. Input samples were normalized to GAPDH, and immunoprecipitated samples were normalized to CNOT3. *p < 0.05, **p < 0.01.

To investigate the biological and biochemical roles of CNOT11, we suppressed CNOT11 expression in HeLa cells using siRNA-mediated knockdown. We first examined the effects of CNOT11 depletion on the expression of other CCR4–NOT complex subunits and on subunit association within the complex. Immunoblot analysis of whole-cell lysates showed that CNOT11 protein levels were markedly reduced in CNOT11-knockdown (CNOT11-KD) cells, whereas the levels of other CCR4–NOT subunits remained largely unchanged compared with control cells (Figure 1A). Densitometric analysis of three independent experiments confirmed the reduction of CNOT11 expression without major alterations in other subunits (Figure 1B).

To examine CCR4–NOT subunit association, we analyzed CNOT3 immunoprecipitates, as immunoprecipitation using an anti-CNOT3 antibody enables enrichment of CCR4–NOT complex-associated proteins (Suzuki et al., 2015). Immunoblot analysis revealed that the association of CNOT10 with the CCR4–NOT complex was significantly reduced in CNOT11-KD cells compared with scramble siRNA–transfected control cells (Figures 1A,B). These results suggest that CNOT11 depletion does not broadly alter the expression of CCR4–NOT subunits but is accompanied by selective changes in complex organization.

### CNOT11 knockdown is associated with changes in autophagy-related markers

**FIGURE 2:**
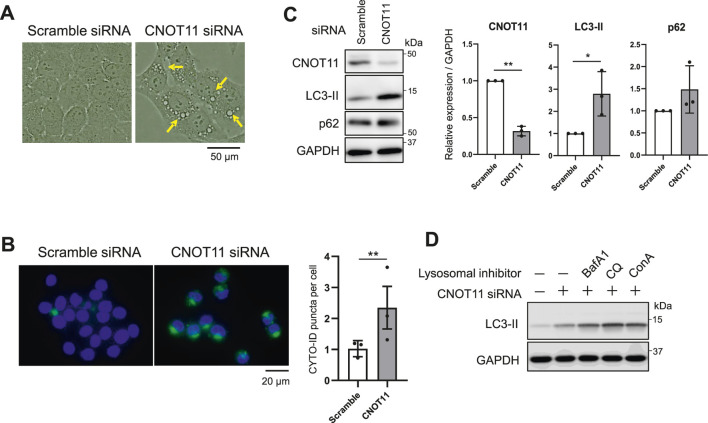
Autophagy-related responses in CNOT11-KD cells **(A)** Phase-contrast images. Cytoplasmic vesicle-like structures are indicated by arrows. **(B)** Cyto-ID® Green (green) and Hoechst 33342 (blue) staining. Representative images and quantification of Cyto-ID–positive puncta per cell are shown. **p < 0.01. **(C)** Immunoblot analysis of CNOT11, LC3, and p62. Band intensities were normalized to GAPDH. *p < 0.05, **p < 0.01. **(D)** Immunoblot analysis of LC3 following lysosomal inhibitor treatment (bafilomycin A1 5 nM, chloroquine 50 nM, concanamycin A 5 nM, 4 h). The blots shown were obtained from the same experiment and processed in parallel.

Phase-contrast microscopy images of CNOT11-KD cells revealed an accumulation of small cytoplasmic vesicle-like structures compared with scramble siRNA–transfected control cells (Figure 2A). Staining with the autophagy-associated vesicle dye Cyto-ID revealed an increased number of punctate structures in CNOT11-KD cells (Figure 2B). Quantification of Cyto-ID–positive puncta per cell confirmed an increase in puncta number in CNOT11-KD cells compared with control cells. Immunoblot analysis demonstrated that LC3-II levels were increased in CNOT11-KD cells relative to control cells (Figure 2C).

To examine LC3-II accumulation after lysosomal inhibitor treatment, cells were treated with bafilomycin A1, chloroquine, or concanamycin A. LC3-II levels were further increased in CNOT11-KD cells following lysosomal inhibitor treatment (Figure 2D), indicating that LC3-II accumulation can be further enhanced under these conditions. These results are consistent with the interpretation that lysosomal degradation is not completely blocked in CNOT11-KD cells.

To assess whether LC3-II accumulation upon CNOT11 depletion is observed beyond HeLa cells, we performed immunoblot analysis in A549 lung cancer cells and HT29 colon cancer cells. CNOT11 depletion increased LC3-II levels in both cell lines (Supplementary File 1), suggesting that this response is not restricted to HeLa cells. Notably, CNOT11 protein expression was markedly lower in BJ normal human fibroblasts compared with the cancer cell lines examined (Supplementary File 2), which provides additional context for focusing mechanistic analyses on cancer cell lines in the present study.

### CNOT11 depletion is associated with altered p62 turnover and autophagy-related gene expression

Although LC3-II accumulation was observed in CNOT11-KD cells, p62 (SQSTM1) protein levels tended to increase, but this change did not reach statistical significance (Figure 2C). Re-examination of transcriptome data confirmed that *SQSTM1* mRNA was significantly upregulated in CNOT11-KD cells (logFC = 0.30, FDR = 2.5 × 10^−10^). Consistent with this finding, transcriptomic analysis revealed coordinated changes in genes related to autophagy initiation, lysosome-associated pathways, mitophagy, and IL-6/JAK-STAT signaling (Supplementary File 3). These findings suggest that the relatively preserved p62 protein levels may, at least in part, reflect transcriptional induction rather than impaired degradation alone.

**FIGURE 3:**
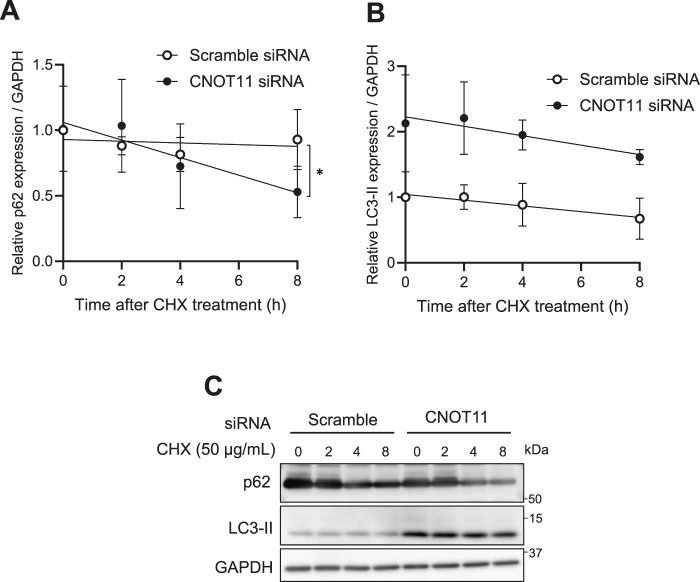
Cycloheximide-based p62 turnover assay in CNOT11-KD cells. **(A)** Quantification of relative p62 protein levels normalized to GAPDH. **(B)** Quantification of relative LC3-II levels normalized to GAPDH. **(C)** Representative immunoblots of p62, LC3, and GAPDH. HeLa cells were transfected with scramble or CNOT11 siRNA for 72 h and then treated with cycloheximide (CHX; 50 μg/mL). Cells were harvested at the indicated time points after CHX addition. Time-course data were analyzed by two-way ANOVA followed by Šídák’s multiple comparisons test. A significant difference between scramble siRNA and CNOT11 siRNA was observed at 8 h in panel **(A)**. *p < 0.05. LC3-II levels remained elevated in CNOT11-KD cells throughout the time course, without a clear time-dependent decrease in either group.

To evaluate p62 protein turnover, cycloheximide (CHX)-based turnover assays were performed. Following CHX treatment, p62 protein levels showed a greater decline in CNOT11-KD cells than in control cells over the time course, with a significant difference observed at 8 h (Figures 3A,C), suggesting that p62 turnover was not impaired by CNOT11 depletion. LC3-II levels remained elevated throughout the time course in CNOT11-KD cells compared with control cells, without a clear time-dependent decrease in either group (Figures 3B,C). These findings suggest that p62 turnover is not impaired under these conditions and are compatible with changes in autophagy-related processes in CNOT11-KD cells, although they do not prove altered autophagic flux.

### CNOT11 knockdown is associated with changes in AMPK/ULK1 signaling

**FIGURE 4:**
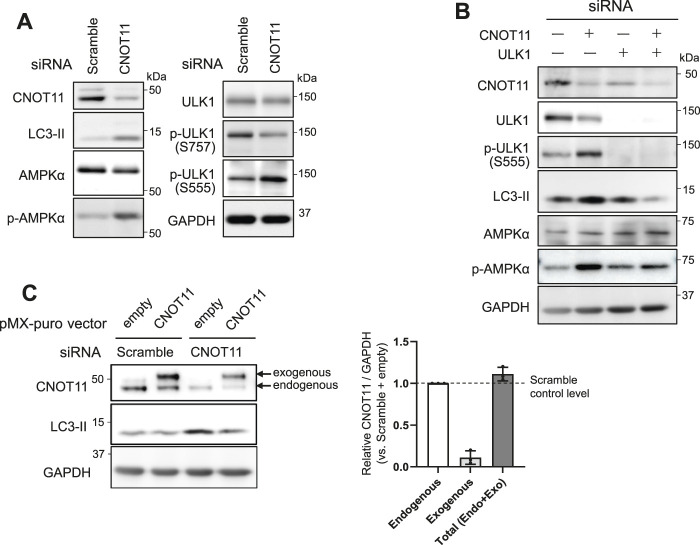
Association of AMPK/ULK1 signaling with autophagy-related responses in CNOT11-KD cells. **(A)** Immunoblot analysis of HeLa cells transfected with scramble or CNOT11 siRNA using the indicated antibodies. **(B)** Immunoblot analysis of HeLa cells co-transfected with CNOT11 siRNA and ULK1 siRNA as indicated. **(C)** Immunoblot analysis of HeLa cells stably expressing siRNA-resistant FLAG-tagged CNOT11 or vector control, transfected with scramble or CNOT11 siRNA. Endogenous and exogenous CNOT11 bands were quantified separately based on their molecular weight difference and normalized to GAPDH. Total represents the sum of endogenous and exogenous CNOT11 levels. All values are expressed relative to the endogenous CNOT11 level in scramble siRNA-transfected empty vector cells (set to 1.0; dashed line). The blots shown were obtained from the same experiment and processed in parallel.

Autophagy initiation is regulated by the ULK1 complex, which is controlled by mTOR and AMPK in response to cellular nutrient and energy status. Under nutrient-rich conditions, mTORC1 phosphorylates ULK1 at Ser757, inhibiting its interaction with AMPK (Klionsky et al., 2021). In contrast, energy stress activates AMPK, which phosphorylates ULK1 at Ser555 to promote autophagy initiation (Kim et al., 2011). We therefore examined the involvement of AMPK and ULK1 signaling in CNOT11-KD cells. Immunoblot analysis showed increased phosphorylation of AMPKα and ULK1 at Ser555, accompanied by reduced phosphorylation of ULK1 at Ser757 in CNOT11-KD cells (Figure 4A). These signaling changes were accompanied by LC3-II accumulation.

Simultaneous knockdown of ULK1 and CNOT11 partially reduced LC3-II levels compared with CNOT11-KD cells, while p-AMPKα levels remained elevated (Figure 4B). To confirm the specificity of the LC3-II accumulation phenotype, siRNA-resistant CNOT11 was reintroduced into CNOT11-KD cells. Re-expression of siRNA-resistant CNOT11 attenuated LC3-II accumulation, whereas vector-transfected CNOT11-KD cells maintained elevated LC3-II levels (Figure 4C), supporting the specificity of the CNOT11 depletion-associated autophagy-related phenotype.

### IL-6/JAK–STAT signaling is associated with LC3-II accumulation in CNOT11-KD cells

**FIGURE 5:**
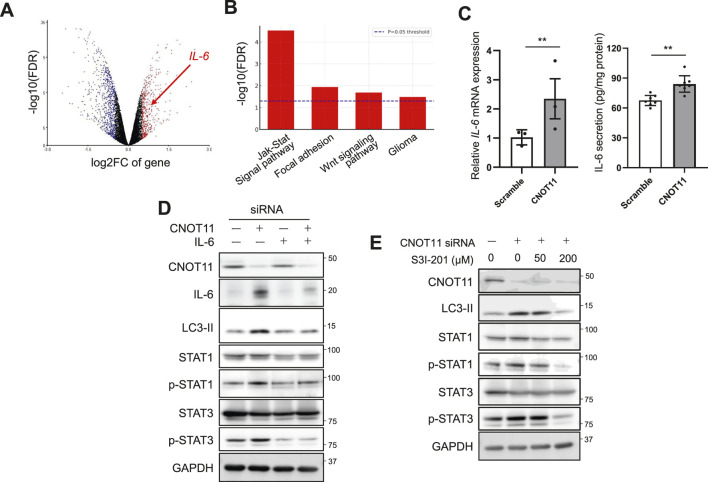
Association of IL-6/JAK–STAT signaling with LC3-II accumulation in CNOT11-KD cells. **(A)** Volcano plot of RNA-seq data comparing CNOT11-KD cells and scramble siRNA-treated control cells. *IL-6* is highlighted. **(B)** Pathway enrichment analysis of differentially expressed genes in CNOT11-KD cells. **(C)** Left: Quantitative RT-PCR analysis of *IL-6* mRNA levels. Right: ELISA analysis of IL-6 secretion in culture medium. **p < 0.01. **(D)** Immunoblot analysis of HeLa cells transfected with the indicated siRNAs targeting CNOT11 and/or IL-6, followed by detection of CNOT11, IL-6, LC3, STAT1, phospho-STAT1, STAT3, phospho-STAT3, and GAPDH. **(E)** Immunoblot analysis following treatment with the STAT3 inhibitor S3I-201 (0, 50, or 200 μM, 6 h). The blots shown were obtained from the same experiment and processed in parallel.

To gain insight into the molecular mechanisms underlying the autophagy-related changes observed in CNOT11-KD cells, we performed RNA-seq analysis to identify global transcriptional changes. Differential expression analysis revealed both upregulated and downregulated genes compared with control cells (Figure 5A), and pathway enrichment analysis identified enrichment of the JAK–STAT signaling pathway (Figure 5B). Quantitative RT-PCR confirmed a significant increase in *IL-6* mRNA expression, and ELISA analysis demonstrated increased IL-6 secretion in the culture medium of CNOT11-KD cells (Figure 5C).

To examine changes in IL-6–related signaling, immunoblot analysis was performed. Intracellular IL-6 protein levels were increased in CNOT11-KD cells. This increase was accompanied by enhanced phosphorylation of STAT1 and STAT3 together with LC3-II accumulation (Figure 5D). To assess whether IL-6 signaling was involved in LC3-II accumulation, simultaneous knockdown of CNOT11 and IL-6 was performed. IL-6 knockdown partially reduced LC3-II levels in CNOT11-KD cells (Figure 5D). Likewise, treatment with the STAT3 inhibitor S3I-201 attenuated LC3-II accumulation (Figure 5E). To further explore signaling changes associated with IL-6 signaling, we additionally examined p38 MAPK activation. Immunoblot analysis showed that phospho-p38 levels were increased in CNOT11-KD cells and were attenuated upon IL-6 co-knockdown (Supplementary File 4), suggesting a possible association between IL-6 signaling and p38 MAPK activation in this context.

### IL-6 upregulation in CNOT11-KD cells is associated with increased transcription

**FIGURE 6:**
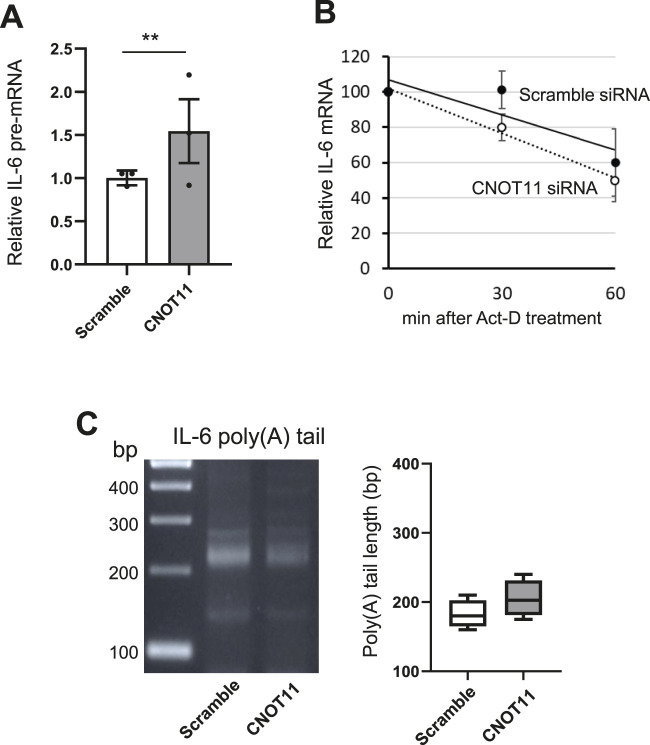
Analysis of IL-6 transcription, mRNA stability, and poly(A) tail length in CNOT11-KD cells. **(A)** Quantitative RT-PCR analysis of unspliced *IL-6* precursor transcripts (*pre-IL-6*). **p < 0.01. **(B)** *IL-6* mRNA stability analysis following actinomycin D (2.5 μg/mL) treatment at the indicated time points. **(C)** Poly(A) tail length analysis of *IL-6* mRNA. Left: Representative PAT assay gel image. Right: Quantification of *IL-6* poly(A) tail length distribution.

We next investigated whether increased transcription or altered mRNA stability contributed to the increased *IL-6* expression observed in CNOT11-KD cells. Quantitative RT-PCR analysis showed a significant increase in unspliced *IL-6* transcripts in CNOT11-KD cells compared with control cells (Figure 6A). To assess whether *IL-6* mRNA stabilization contributed to the elevated *IL-6* expression, we measured mRNA decay following actinomycin D treatment. No clear difference in *IL-6* mRNA decay kinetics was observed between control and CNOT11-KD cells (Figure 6B). Poly(A) tail length was analyzed using a PAT assay. The distribution of *IL-6* poly(A) tail length was comparable between control and CNOT11-KD cells (Figure 6C). These results suggest that the increased IL-6 expression in CNOT11-KD cells is not primarily explained by changes in mRNA stability or poly(A) tail length. To provide additional context for the possible involvement of CNOT11 in transcription-related processes, we examined the subcellular localization of endogenous CNOT11 by immunofluorescence analysis. Quantitative analysis of nuclear-to-cytoplasmic (N/C) fluorescence intensity ratios revealed that CNOT11 was preferentially localized in the nucleus in control cells (N/C ratio = 2.08 ± 0.17), and this nuclear enrichment was significantly reduced following CNOT11 depletion (N/C ratio = 1.16 ± 0.10, p < 0.001; Supplementary File 4). These findings suggest that endogenous CNOT11 is preferentially localized to the nucleus in HeLa cells, consistent with the possibility that CNOT11 may be linked to transcription-related processes.

### Working model of CNOT11 depletion-associated autophagy-related responses and cytokine signaling

**FIGURE 7:**
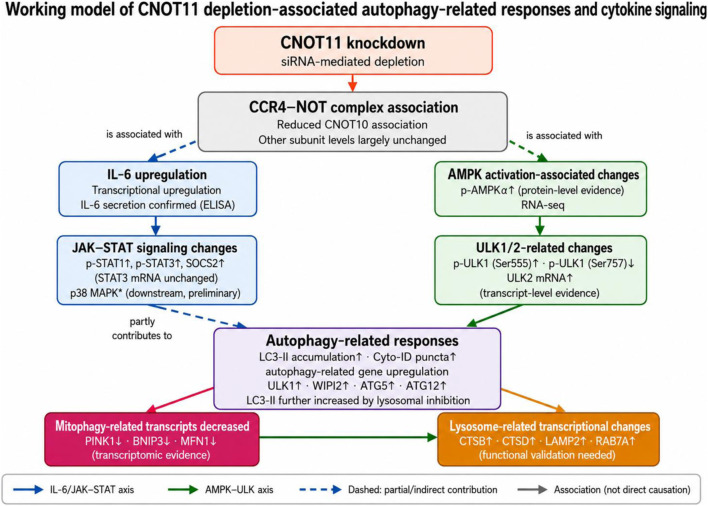
Working model of CNOT11 depletion-associated autophagy-related responses and cytokine signaling. CNOT11 depletion is associated with altered CCR4–NOT complex organization, including reduced association of CNOT10, and with changes in IL-6/JAK–STAT and AMPK–ULK1 signaling pathways. These signaling changes are associated with LC3-II accumulation, increased Cyto-ID–positive puncta, further LC3-II accumulation upon lysosomal inhibitor treatment, and transcriptional upregulation of autophagy-related genes. The mechanistic relationship between IL-6/JAK–STAT and AMPK–ULK1 signaling in CNOT11-depleted cells remains to be determined. Mitophagy-related transcripts were decreased, and lysosome-related transcriptional changes were identified; however, functional validation of lysosomal activity remains to be performed. Solid arrows indicate experimentally supported associations, dashed arrows indicate indirect or partial functional contributions, and gray dashed arrows indicate associations not directly established as causal relationships. General note: *p < 0.05, **p < 0.01 versus scramble siRNA–transfected control (except Figure 3A, which was analyzed by two-way ANOVA; see legend for details). For immunoblot quantification analyses in Figures 1, 2C, 3, 4, band intensities were normalized to the corresponding scramble control within each independent experiment, which was set to 1.0. Therefore, variation is not shown for scramble control bars in these analyses. Non-immunoblot assays were presented as raw measured values.

To integrate the molecular and experimental findings obtained in this study, we constructed a working model summarizing the changes associated with CNOT11 depletion (Figure 7). CNOT11 knockdown was associated with altered CCR4–NOT complex organization, including reduced association of CNOT10, and with changes in two signaling pathways. One signaling branch associated with CNOT11 depletion includes IL-6 upregulation and increased phosphorylation of STAT1 and STAT3. CNOT11 depletion was also associated with changes in the AMPK–ULK1 pathway, including increased phosphorylation of AMPKα and ULK1 at Ser555 and decreased phosphorylation of ULK1 at Ser757. The mechanistic relationship between these two pathways remains to be determined. These signaling changes were associated with LC3-II accumulation, autophagy-related transcriptional changes, and reduced expression of mitophagy-related genes.

## Discussion

In this study, we examined the role of CNOT11, a component of the CCR4–NOT deadenylase complex, in the regulation of cellular stress responses. Depletion of CNOT11 reduced the association of CNOT10 with the complex, suggesting that CNOT11 contributes to subunit association within the CCR4–NOT complex. CNOT11 knockdown was associated with autophagy-related responses and changes in IL-6/JAK–STAT signaling. Similar stress-associated phenotypes have been reported following depletion of other CCR4–NOT subunits, including CNOT3 (Suzuki et al., 2015). Notably, the increase in IL-6 expression was not attributable to changes in mRNA stability or poly(A) tail length, suggesting that CNOT11 may influence certain cellular responses through mechanisms distinct from bulk deadenylation-dependent mRNA decay (Collart, 2016; Hagkarim and Grand, 2020).

Our data suggest that CNOT11 depletion is associated with LC3-II accumulation that can be further enhanced by lysosomal inhibitor treatment, rather than a complete blockade of lysosomal degradation. Although p62/SQSTM1 is a well-known autophagy substrate degraded through autophagic pathways (Bjørkøy et al., 2005), p62 protein levels showed a tendency to increase without reaching statistical significance. Re-examination of transcriptome data confirmed significant transcriptional upregulation of SQSTM1 as described above, and CHX-based turnover assays suggested that p62 protein turnover was not impaired and may be increased in CNOT11-KD cells (Figure 3A). The absence of a statistically significant change in p62 protein levels may therefore reflect a dynamic balance between transcriptional upregulation of SQSTM1, which can be induced by stress-responsive pathways such as NRF2 signaling (Jain et al., 2010), and increased turnover of p62 protein in CNOT11-KD cells. p62/SQSTM1 functions as a key adaptor linking autophagy and ubiquitin-dependent protein degradation pathways (Liu et al., 2016). At the signaling level, increased phosphorylation of AMPKα and ULK1 at Ser555, together with decreased phosphorylation of ULK1 at Ser757, indicates changes in the AMPK–ULK1 axis associated with LC3-II accumulation. AMPK and mTOR are known to coordinately regulate autophagy through direct phosphorylation of ULK1 (Kim et al., 2011). AMPK-mediated phosphorylation of ULK1 has been reported to link cellular energy sensing to autophagy-related processes, including mitophagy (Egan et al., 2011). However, the molecular mechanism linking altered CCR4–NOT subunit association to AMPK activation remains unclear.

Cytokine signaling pathways have been implicated in the regulation of autophagy in cancer cells (Johnson et al., 2018). IL-6 emerged as a signaling component associated with LC3-II accumulation in CNOT11-KD cells, as IL-6 knockdown or STAT3 inhibition attenuated this phenotype. These findings support a partial involvement of IL-6/JAK–STAT signaling in the CNOT11 depletion-associated phenotype, although its precise causal position remains to be further clarified as discussed in the Limitations section. In addition, canonical STAT3 target genes associated with autophagy, such as BNIP3, DRAM1, and BCL2, were not upregulated in our dataset. Thus, the mechanism linking IL-6/JAK–STAT signaling to LC3-II accumulation remains unclear and may involve additional signaling pathways, including possible interactions with the AMPK–ULK1 axis (Johnson et al., 2018; Xu et al., 2022).

The upregulation of IL-6 mRNA was not explained by detectable changes in mRNA stability or poly(A) tail length, suggesting that increased transcription contributes to IL-6 upregulation in CNOT11-KD cells. To further explore potential upstream mechanisms, we examined phosphorylated NF-κB p65 levels by immunoblot analysis. Phosphorylated NF-κB p65 levels were not detectably altered in CNOT11-KD cells (Supplementary File 4), suggesting that canonical NF-κB activation is unlikely to be the primary driver of IL-6 induction in this context. Thus, the precise upstream mechanism underlying IL-6 induction remains unclear. We also observed that phospho-p38 MAPK levels were increased in CNOT11-KD cells and attenuated upon IL-6 co-knockdown, suggesting a possible association between IL-6 signaling and p38 MAPK activation in this context (Supplementary File 4).

Furthermore, the nuclear-predominant localization of endogenous CNOT11 observed in control cells (Supplementary File 4) raises the possibility that CNOT11 may be linked to transcription-related processes beyond its canonical role in cytoplasmic mRNA decay, consistent with previous reports demonstrating direct roles of the CCR4–NOT complex in transcription elongation (Collart and Struhl, 1993; Kruk et al., 2011). It should also be noted that HeLa cells have impaired p53 function (Hietanen et al., 2000), which may influence autophagy regulation and IL-6/JAK–STAT signaling in a context-dependent manner. Importantly, LC3-II accumulation following CNOT11 depletion was also observed in HT29 and A549 cells, suggesting that this phenotype is not restricted to the p53-impaired HeLa cell context. In addition, public RNA-seq database analyses did not reveal a statistically significant association between CNOT11 expression and overall survival in the cancer types examined, suggesting that the prognostic relevance of CNOT11 may be context-dependent. This lack of statistical significance may also reflect potential confounding factors inherent to public clinical datasets, including differences in tumor stage, prior or subsequent treatment history, molecular subtype, sample size, and cohort heterogeneity. Therefore, larger and more clinically annotated cohorts will be required to determine whether CNOT11 expression has prognostic value in specific cancer contexts. Nevertheless, CNOT11 protein was detected across multiple cancer cell lines with varying expression levels (Supplementary File 2), supporting the possibility that CNOT11 may have broader relevance in cancer biology.

Taken together, our results suggest that CNOT11 depletion is associated with altered CCR4–NOT complex organization, cytokine signaling, and autophagy-related responses, and support a potential role for CNOT11 as a modulator of stress-response networks in cancer cells.

### Limitations

This study has several limitations that should be considered. First, although CNOT11 depletion was associated with LC3-II accumulation, increased Cyto-ID–positive puncta, altered p62 turnover, and further LC3-II accumulation upon lysosomal inhibitor treatment, definitive assessment of autophagic flux will require gold-standard approaches such as tandem fluorescent LC3 reporters, electron microscopy, or long-lived protein degradation assays. Second, although IL-6 secretion was directly measured by ELISA and IL-6 knockdown or STAT3 inhibition attenuated LC3-II accumulation in CNOT11-KD cells, recombinant IL-6 rescue, IL-6 neutralization, and conditioned-medium transfer experiments were not performed. Therefore, IL-6/JAK–STAT signaling should be interpreted as a partial contributor rather than a definitive upstream mediator. Third, the mechanistic relationships among altered CCR4–NOT complex organization, IL-6 induction, AMPK–ULK1 signaling, p38 MAPK activation, and LC3-II accumulation remain unresolved. In particular, ChIP-based validation will be required to determine whether CCR4–NOT components are directly involved in transcriptional regulation of the IL-6 promoter. Fourth, most mechanistic experiments were performed in HeLa cells, which have impaired p53 function. Although LC3-II accumulation following CNOT11 depletion was also observed in HT29 and A549 cells, the downstream mechanisms in these additional cell lines remain to be determined. Finally, public Kaplan-Meier analyses did not reveal a statistically significant association between CNOT11 expression and overall survival in the cancer types examined, which may reflect potential confounding factors such as tumor stage, treatment history, molecular subtype, sample size, and cohort heterogeneity. Larger and more clinically annotated cohorts will be required to clarify the prognostic relevance of CNOT11 in specific cancer contexts.

## Conclusion

This study suggests that CNOT11, a vertebrate-specific subunit of the CCR4–NOT complex, may be involved in modulating autophagy-related responses and cytokine signaling in cancer cells, with partial contribution from IL-6/JAK–STAT signaling. These findings highlight a previously unrecognized link between CCR4–NOT complex organization and cellular stress-response networks, and provide a foundation for future studies aimed at defining the precise molecular mechanisms by which CNOT11 links RNA regulatory and signaling processes in cancer cells.

## Materials and methods

### Cells and reagents

HeLa (RCB0007) cells were obtained from the RIKEN BioResource Research Center (RIKEN BRC, Tsukuba, Japan). Bafilomycin A1 and the STAT3 inhibitor S3I-201 were purchased from Sigma-Aldrich (St. Louis, MO, USA). Chloroquine and concanamycin A were obtained from Tocris Bioscience (Bristol, UK).

### Antibodies

Antibodies against CNOT1, CNOT2, CNOT3, CNOT6, CNOT6L, CNOT8, CNOT9, and CNOT10 were used as previously described (Suzuki et al., 2015). The antibody against CNOT11 (HPA069823) was obtained from Atlas Antibodies AB (Bromma, Sweden). Antibodies against GAPDH (#2118), AMPKα (#2603), phospho-AMPKα (Thr172; #2535), ULK1 (#8054), phospho-ULK1 (Ser555; #5869), IL-6 (#12153), STAT1 (#9172), phospho-STAT1 (Tyr701; #7649), STAT3 (#4904), phospho-STAT3 (Tyr705; #9145), cleaved PARP (#9541), p38 MAPK (#8690), phospho-p38 MAPK (Thr180/Tyr182; #4511), phospho-NF-κB (#3033), phospho-eIF2α (#9721) and SQSTM1/p62 (#5114) were obtained from Cell Signaling Technology (Danvers, MA, USA). The LC3 antibody (PM036) was obtained from MBL International (Woburn, MA, USA).

### Cell culture and siRNA transfection

HeLa cells were cultured in Dulbecco’s Modified Eagle’s Medium (DMEM; Fujifilm Wako, Osaka, Japan) supplemented with 10% fetal bovine serum (FBS; Nichirei Corporation, Tokyo, Japan) and 100 U/mL penicillin–streptomycin (Thermo Fisher Scientific, Waltham, MA, USA) at 37 °C in a humidified atmosphere containing 5% CO_2_. For siRNA-mediated knockdown, 100 pmol of double-stranded siRNA was transfected into 5 × 10^5^ cells using Lipofectamine RNAiMAX (Thermo Fisher Scientific). Unless otherwise indicated, the same siRNA transfection conditions were used for A549 and HT29 cells. The siRNA sequences used were as follows: scramble, 5′-UUC​UCC​GAA​CGU​GUC​ACG​UTT-3′ and 5′-ACG​UGA​CAC​GUU​CGG​AGA​ATT-3′; *CNOT11*, 5′-CCG​AAC​GCC​AAU​CUG​AAU​UGC-3′ and 5′-AAU​UCA​GAU​UGG​CGU​UCG​GCC-3′; *ULK1*, 5′-GCA​CAG​AGA​CCG​UGG​GCA​ATT-3′ and 5′-UUG​CCC​ACG​GUC​UCU​GUG​CTT-3′. For IL-6 knockdown, a Silencer™ pre-designed siRNA targeting human *IL6* (ID: 144,576; Cat# AM16708; Thermo Fisher Scientific) was used. Cells were analyzed 72 h after transfection.

### Analysis of autophagy-related markers and lysosomal inhibitor treatment

Autophagy-related responses were assessed in HeLa cells 72 h after siRNA transfection. For detection of autophagy-associated vesicular structures, cells were incubated with CYTO-ID® Green Detection Reagent (1:1000 dilution; Enzo Life Sciences, Farmingdale, NY, USA) for 30 min at 37 °C in the dark. Nuclei were counterstained with Hoechst 33,342, and cells were washed twice with the assay buffer provided by the manufacturer. Fluorescence images were acquired using a laser scanning confocal microscope (TCS SPE; Leica Microsystems, Wetzlar, Germany) under identical imaging settings across all experimental groups. For quantification, Cyto-ID–positive puncta were defined as discrete fluorescent foci distinguishable from diffuse cytoplasmic background. The number of Cyto-ID–positive puncta per cell was manually counted in at least three independent fields, and at least 30 cells were analyzed per condition. The average number of puncta per cell was calculated for each condition. For lysosomal inhibitor-based analysis of LC3-II accumulation, cells were treated with bafilomycin A1 (5 nM), chloroquine (50 nM), or concanamycin A (5 nM) during the final 4 h of incubation. After treatment, cells were subjected to immunoblotting to analyze LC3-II protein levels, and parallel samples were examined by phase-contrast microscopy.

### Immunoprecipitation and immunoblot

Cells were washed with PBS and lysed in TNE buffer containing 50 mM Tris-HCl (pH 7.5), 150 mM NaCl, 1 mM EDTA, 1 mM phenylmethylsulfonyl fluoride, 10 mM NaF, 10 mM β-glycerophosphate, and 1% Nonidet P-40. Cell lysates were immunoprecipitated using 1 µg of mouse monoclonal anti-CNOT3 antibody and Dynabeads Protein G (Thermo Fisher Scientific) overnight at 4 °C with rotation. Normal mouse IgG was used in parallel as a negative control for immunoprecipitation. Immunoblot detection was performed using Immobilon Western Chemiluminescent HRP Substrate (Merck Millipore), and chemiluminescent signals were visualized using an Amersham Imager 680 (GE Healthcare). Band intensities were quantified using ImageJ software. Band intensities of total lysates were normalized to GAPDH unless otherwise indicated, whereas band intensities of immunoprecipitated samples were normalized to CNOT3 levels to account for differences in immunoprecipitation efficiency. Values were expressed relative to control samples.

### Virus infection

Retroviruses were produced by transfecting Plat-E packaging cells with 2 µg of pMXs-puro vectors encoding CNOT11 cDNA using TransIT-LT1 transfection reagent (Mirus Bio LLC). Forty-eight hours later, supernatants were collected, filtered, supplemented with polybrene (5 μg/mL), and used to infect ecotropic receptor–expressing HeLa cells. Two days after retroviral infection, cells were trypsinized, diluted, and cultured in the presence of puromycin (1 μg/mL) for an additional 3 days to select infected cell populations.

### Reverse transcriptase PCR assays and mRNA half-life measurement

Total RNA was extracted using Isogen reagent (Nippon Gene Co., Ltd., Toyama, Japan). Complementary DNA (cDNA) was synthesized from 0.5–1 µg of total RNA using PrimeScript II reverse transcriptase (Takara Bio Inc., Shiga, Japan). Quantitative reverse transcription PCR (qRT-PCR) was performed using a QuantStudio™ 5 Real-Time PCR System (Applied Biosystems, Foster City, CA, USA) and FastStart Universal SYBR Green Master Mix (Merck). Gene expression levels were normalized to *GAPDH*. For mRNA stability analysis, cells were treated with actinomycin D (2.5 μg/mL; Sigma-Aldrich) 72 h after siRNA transfection, and RNA was collected at 0, 30, and 60 min following treatment.

### Poly(A) tail assay

Poly(A) tail lengths of mRNAs were analyzed using the Poly(A) Tail-Length Assay Kit (Thermo Fisher Scientific) with minor modifications. Total RNA (1 µg) was incubated with poly(A) polymerase to add a GI tail, and cDNA was synthesized using a PAT universal primer and reverse transcriptase. PCR amplification was performed using gene-specific primers, the PAT universal primer, and HotStart-IT® Taq DNA Polymerase (Affymetrix, Santa Clara, CA, USA), and products were separated by agarose gel electrophoresis.

### RNA sequencing and analysis

RNA-seq was performed on total RNA from HeLa cells by the DNA Sequencing Section at OIST using the TruSeq Stranded mRNA Library Prep Kit for NeoPrep (Cat# NP-202–1001; Illumina Inc., San Diego, CA, USA) with 100 ng input RNA. Paired-end sequencing (150 bp × 2) was performed on the HiSeq 3000/4000 system (Illumina Inc.). Reads were mapped and analyzed using Strand NGS software (Strand Life Sciences, Bangalore, India), and transcript abundance was quantified as FPKM. Genes with FDR <0.05 were considered significantly differentially expressed. Pathway and Gene Ontology enrichment analyses were performed using iDEP.96 (http://bioinformatics.sdstate.edu/idep96/) and DAVID Bioinformatics Resources 6.8 (https://david.ncifcrf.gov), respectively. RNA-seq data have been deposited in GEO under accession number GSE306607.

### IL-6 ELISA

Culture supernatants were collected from HeLa cells 72 h after siRNA transfection and clarified by centrifugation at 1,000 × g for 5 min. IL-6 concentrations were measured using a human IL-6 ELISA kit (AuthentiKine™ Human IL-6 ELISA Kit; Cosmo Bio, Tokyo, Japan) and determined from a standard curve.

### Cycloheximide-based p62 turnover assay

HeLa cells transfected with scramble or CNOT11 siRNA were treated with cycloheximide (CHX; 50 μg/mL; Sigma-Aldrich) at 72 h post-transfection and harvested at 0, 2, 4, and 8 h after CHX addition. Cell lysates were analyzed by immunoblotting using antibodies against p62, LC3, and GAPDH.

### IL-6 and STAT3 perturbation assays

For STAT3 inhibition, cells were treated with S3I-201 (50 or 200 µM) for 6 h prior to harvesting. For IL-6 knockdown, CNOT11 and IL-6 siRNAs were simultaneously transfected using Lipofectamine RNAiMAX. LC3-II protein levels were analyzed by immunoblotting as described above.

### Immunofluorescence analysis

Cells were fixed with 4% paraformaldehyde and permeabilized with 0.1% Triton X-100 in PBS. After blocking, cells were incubated with anti-CNOT11 antibody overnight at 4 °C, followed by Alexa Fluor 488–conjugated secondary antibody. Nuclei were counterstained with DAPI. Images were acquired using a Leica TCS SPE confocal microscope, and nuclear-to-cytoplasmic fluorescence intensity ratios were calculated using ImageJ software.

### Statistical analysis

Statistical analyses were performed using GraphPad Prism. Comparisons between two groups were performed using unpaired two-tailed Student’s t-test. Time-course experiments involving two factors, siRNA treatment and time, were analyzed using two-way ANOVA followed by Šídák’s multiple comparisons test when comparisons between treatments at individual time points were performed. Data are presented as mean ± SD from three independent biological replicates (n = 3) unless otherwise stated. A p-value of <0.05 was considered statistically significant.

## Data Availability

The datasets presented in this study can be found in online repositories. The names of the repository/repositories and accession number(s) can be found in the article/Supplementary Material.

## Supplementary material

Supplementary File 1 LC3-II accumulation following CNOT11 depletion in multiple cancer cell lines. HeLa, HT29, and A549 cells were transfected with scramble or CNOT11 siRNA and analyzed by immunoblotting for CNOT11, LC3, and GAPDH. Left: Representative immunoblots. Right: Quantification of LC3-II levels normalized to GAPDH. **p < 0.01.

Supplementary File 2 CNOT11 protein expression across multiple cancer cell lines and Kaplan–Meier survival analysis. **(A)** Immunoblot analysis of CNOT11 expression in BJ fibroblasts and the indicated cancer cell lines. **(B)** Kaplan–Meier survival analysis using the Kaplan–Meier Plotter pan-cancer RNA-seq database. Hazard ratios (HRs) and log-rank p-values are shown.

Supplementary File 3 RNA-seq expression changes of autophagy- and IL-6/JAK-STAT-related genes in CNOT11-KD HeLa cells. Left: Bar plot showing log2 fold changes. Right: Heatmap showing expression patterns of the same gene set. *FDR <0.05, **FDR <0.01, ***FDR <0.001.

Supplementary File 4 Supplementary analyses of p38 MAPK activation, CNOT11 localization, and stress-related signaling markers. **(A)** Immunoblot analysis of HeLa cells transfected with the indicated combinations of CNOT11 siRNA and IL-6 siRNA for CNOT11, IL-6, p38, phospho-p38, and Tubulin. **(B)** Immunofluorescence analysis of endogenous CNOT11 localization in control and CNOT11-KD HeLa cells stained with anti-CNOT11 antibody (green) and DAPI (gray). The marked reduction of CNOT11 signal in CNOT11-KD cells supports antibody specificity. Quantification of nuclear-to-cytoplasmic fluorescence intensity ratios is shown. **p < 0.01. **(C)** Immunoblot analysis of phospho-NF-κB and phospho-eIF2α in HeLa cells transfected with scramble or CNOT11 siRNA. GAPDH was used as a loading control.
