# Supplementary figures and images for: CNOT11 depletion is associated with autophagy-related responses and IL-6–JAK–STAT signaling in cancer cells
Source: Front Cell Dev Biol. 2026 Jul 17;14:1854333. doi: 10.3389/fcell.2026.1854333 (PMC13424701; doi:10.3389/fcell.2026.1854333)

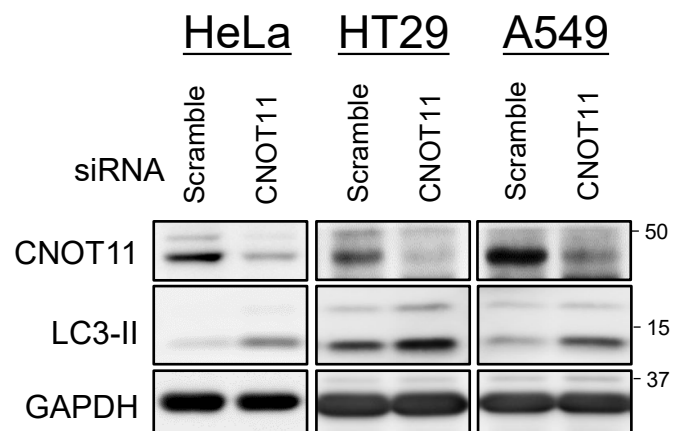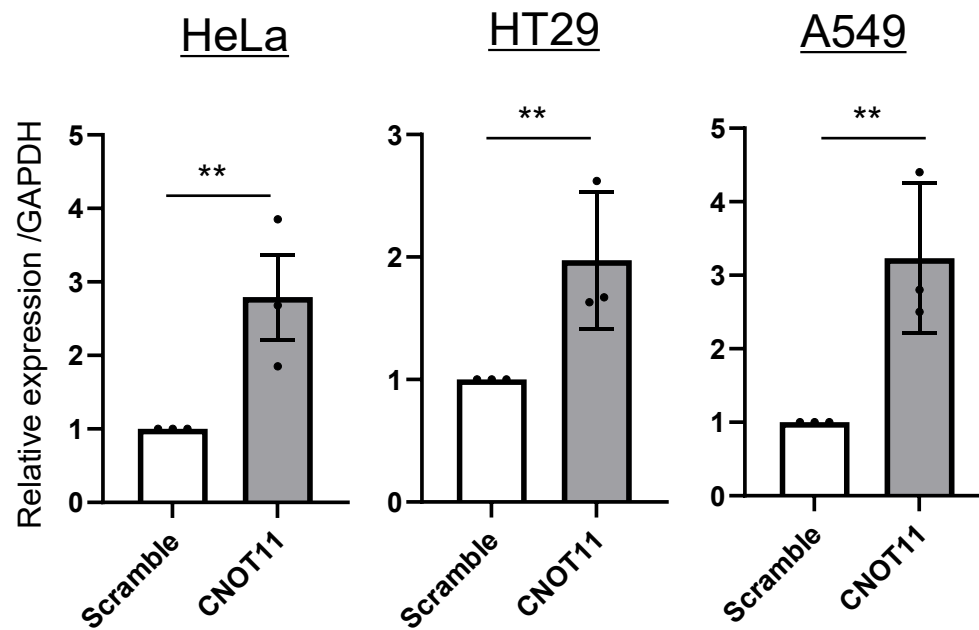

Supplement: Supplementary file 1 [file Supplementaryfile1.pdf]

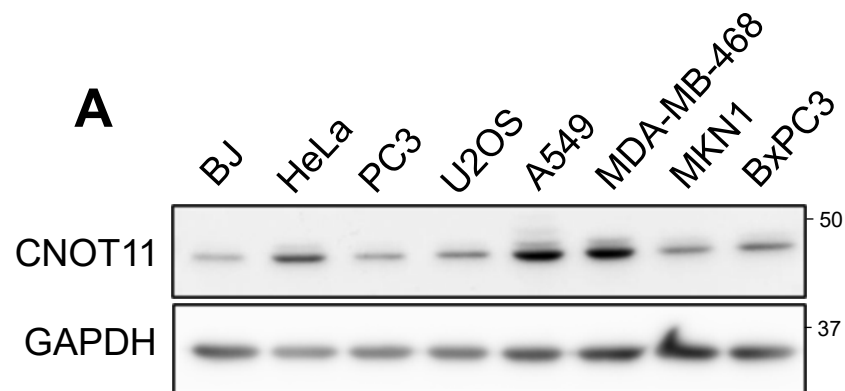

**B**

Cervical squamous cell carcinoma

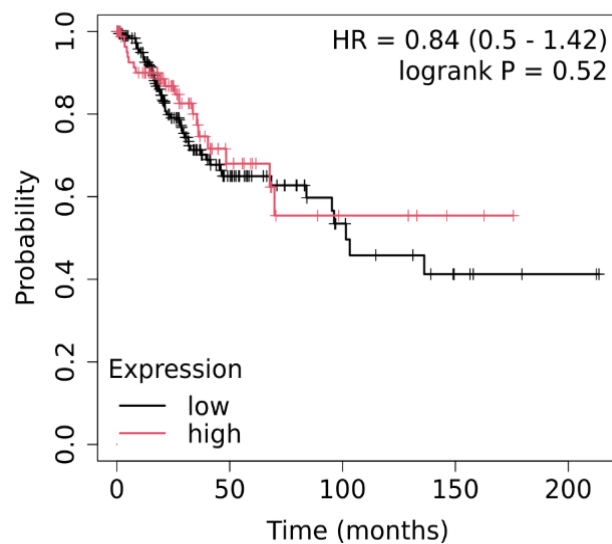

Lung adenocarcinoma

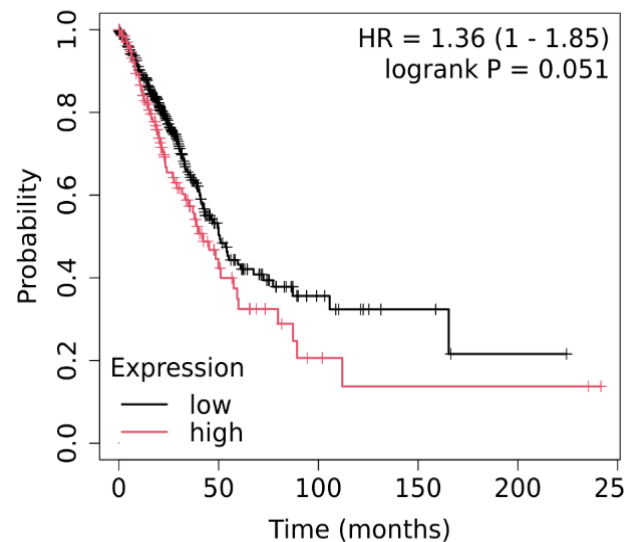

Rectum adenocarcinoma

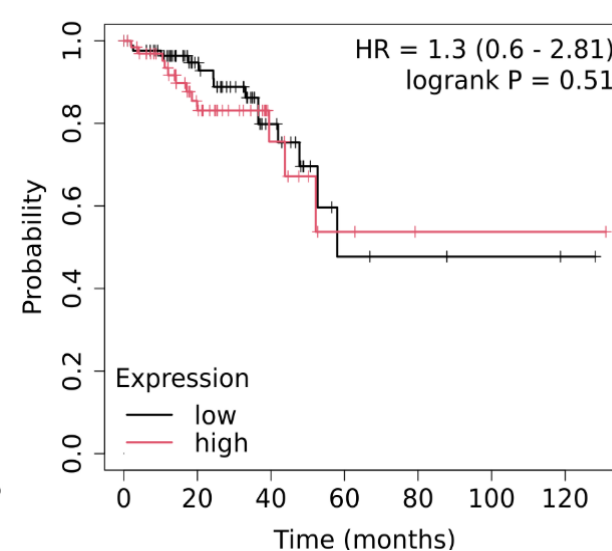

Supplement: Supplementary file 2 [file Supplementaryfile2.pdf]

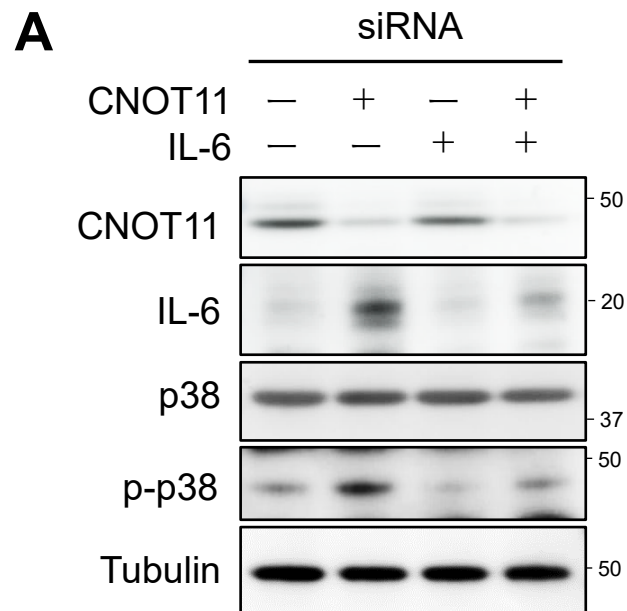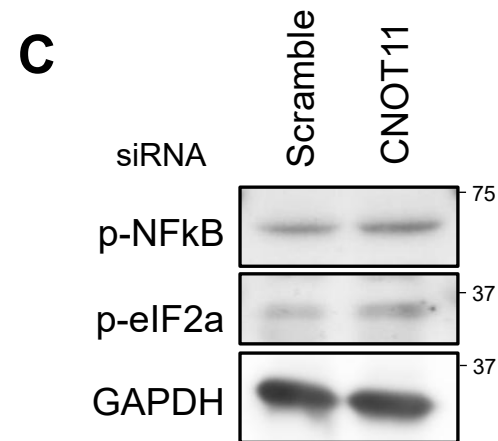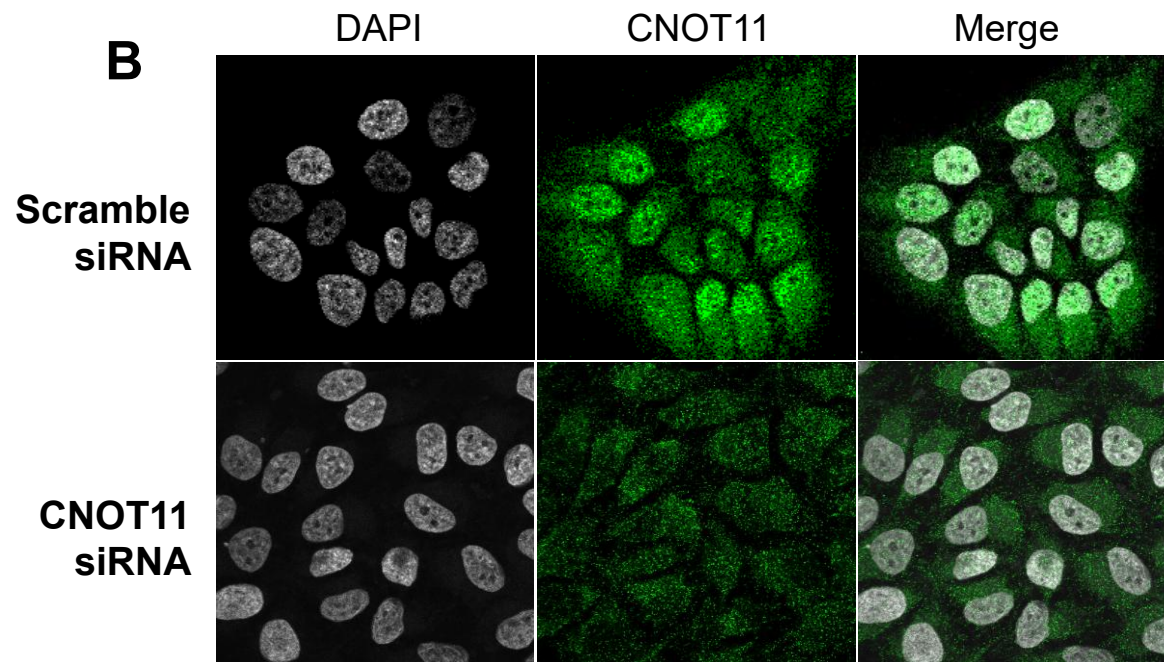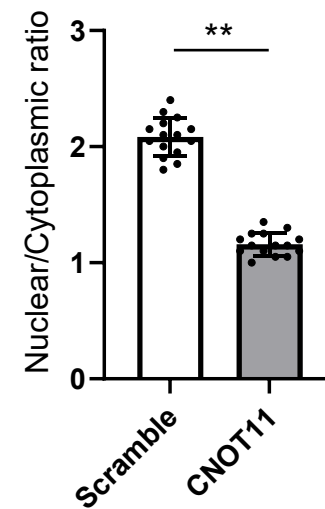

Supplement: Supplementary file 3 [file Supplementaryfile4.pdf]

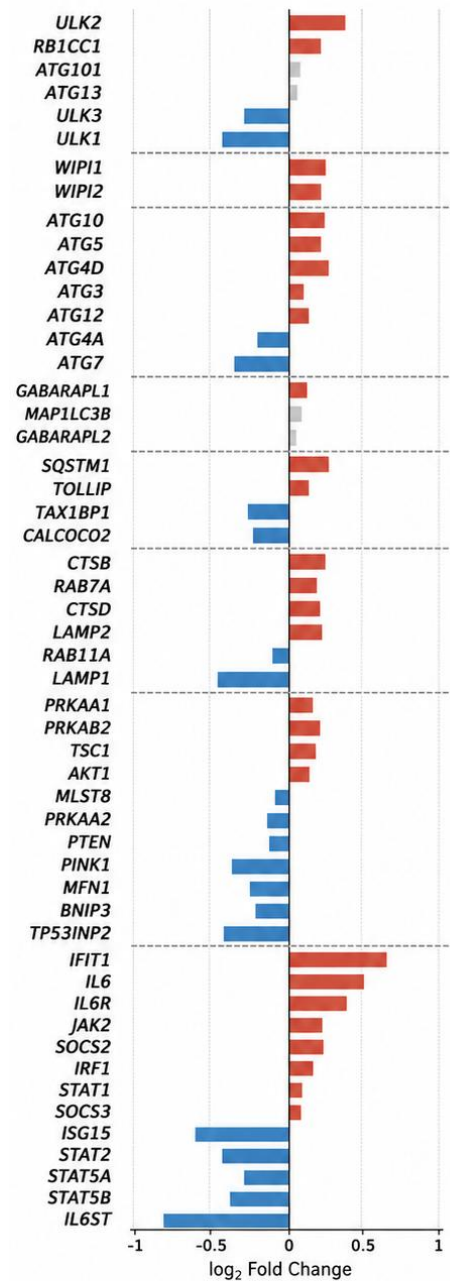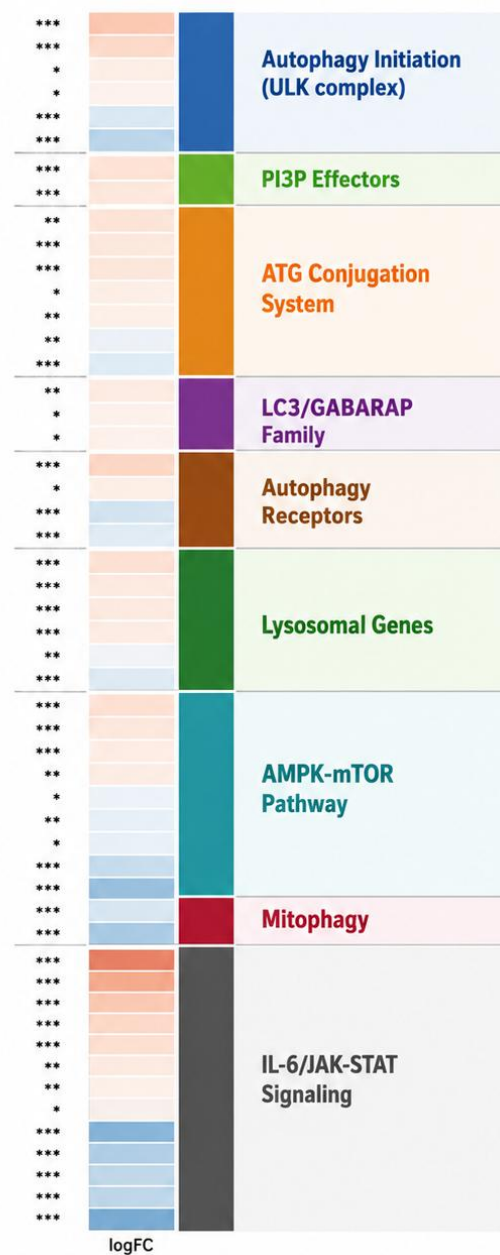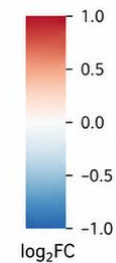

\* FDR < 0.05    \*\* FDR < 0.01    \*\*\* FDR < 0.001

Upregulated (FDR < 0.05)    Downregulated (FDR < 0.05)    Not significant

Supplement: Supplementary file 4 [file Supplementaryfile3.pdf]
